# Generation of Polymer Nanocomposites through Shear-Driven Aggregation of Binary Colloids

**DOI:** 10.3390/polym9110619

**Published:** 2017-11-15

**Authors:** Xinxin Sheng, Li Zhang, Hua Wu

**Affiliations:** 1 Department of Polymeric Materials and Engineering, School of Materials and Energy, Guangdong University of Technology, Guangzhou 510006, China; xinxin.sheng@gdut.edu.cn (X.S.); lizhang@gdut.edu.cn (L.Z.); 2 Institute for Chemical and Bioengineering, Department of Chemistry and Applied Biosciences, ETH Zurich, 8093 Zurich, Switzerland

**Keywords:** polymer nanocomposite, binary colloid, shear-driven, aggregation, gelation

## Abstract

Design of polymer nanocomposites has been an intense research topic in recent decades because hybrid nanomaterials are widely used in many fields. Throughout their development, there has often been a challenging issue how one can uniformly distribute nanoparticles (NPs) in a polymer matrix, avoiding their agglomeration. In this short review, we first introduce the theory of colloidal aggregation/gelation purely based on intense shear forces. Then, we illustrate a methodology for preparing polymer nanocomposites where the NPs (as fillers) are uniformly and randomly distributed inside a matrix of polymer NPs, based on intense shear-driven aggregation of binary colloids, without using any additives. Its feasibility has been demonstrated using two stable binary colloids composed of (1) poly-methyl methacrylate fillers and polystyrene NPs, and (2) graphene oxide sheets (fillers) and poly-vinylidene fluoride NPs. The mechanism leading to capturing and distribution of the fillers inside the polymer NP matrix has been illustrated, and the advantages of the proposed methodology compared with the other common methods are also discussed.

## 1. Introduction

Nanocomposite materials have been widely applied to almost all fields of technology [1], particularly in biomedicine [2], dental and bone implants [3], therapeutics delivery, diagnostics, and treatment [4,5], membrane performance enhancement [6,7,8], coating industry [9,10,11], solid-state lighting, and photovoltaic devices [12,13]. Among them, imbedding nanoparticles (NPs) into polymer matrices to form polymer matrix nanocomposites can improve thermal, mechanical, electric, or optical properties of the polymers [1,14]. In those applications, it is especially important to uniformly and randomly distribute the NPs, as fillers, inside the polymer matrix, while avoiding aggregation among the NPs [15]. Different strategies have been developed in the literature, and among them, the following three are dominating in practical applications: solution mixing [16,17,18,19,20,21], melt compounding [16,19,22,23,24,25], and in situ polymerization [19,26,27,28,29]. Solution mixing is considered to be an effective technique for preparation of polymer nanocomposites, and it disperses fillers into a polymer matrix by simple mechanical stirring or high shear mixing [18,30]. This process often requires a large amount of organic solvent, except for water-based solutions [20,21], and the removal of the residual solvent often becomes crucial. In addition, fillers often re-aggregate during the process of slow solvent evaporation, and it is thus necessary to modify the NP surface to improve the compatibility between NPs and polymer matrix. In the case of melt blending, fillers are mixed with a polymer matrix at the molten state by using mixing equipment such as an extruder, internal mixer, and two-roll mill [22,31]. This process often requires high shear forces to fully mix the molten polymers and fillers, and no solvent is used during the process, making it an eco-friendly method for large scale production of polymer nanocomposites. However, at the molten state the high viscosity of the polymer may lead to poor dispersion of fillers [32,33]. In situ polymerization is another effective way to prepare uniformly distributed fillers inside a polymer matrix. In this process, the fillers are first uniformly dispersed in monomer solution in the presence of an initiator [26,29,34,35,36,37]. Initiated by radiation or thermal energy, in situ polymerization takes place, conferring strong interfacial interactions between the polymer chains and the fillers [29]. Compared with solution mixing and melt blending methods, in situ polymerization can obtain improved compatibility between fillers and polymer, thus better dispersion properties. Kim et al. [19] fabricated graphene-based polyurethane nanocomposites via melt blending, solution mixing, and in situ polymerization, the comparison of the results shows that melt blending would lead to the fillers to re-aggregate, while the other two processes could result in better dispersion of fillers throughout the polymer matrix.

Another important strategy for generation of nanocomposites is to use a colloidal route—Colloidal aggregation. In this way, the applied polymer is initially also in the form of NPs, thus, we have NPs, A and B, forming a binary colloidal system. Binary colloids have received great attention in recent years due to their potential applications in many industrial processes. Aggregation of binary colloids with different types, sizes, or properties has been found to be crucial in the fields such as waste water treatment [38,39], mineral flotation processes [40,41,42,43], cells and DNA analysis [44,45], and composite materials fabrication [46,47,48,49,50,51]. If A and B possess opposite charges, their electrostatic attraction can cause A-B aggregation, typically referred to as hetero-aggregation [52,53,54]. When the size of A and B is similar, the clusters formed from their aggregation are of irregular shape and low fractal dimension. Since the aggregation induced by electrostatic attraction is extremely fast, it is difficult to obtain uniform distribution of A and B within the clusters when the particle concentration is high. When B is much smaller than A, B can attach onto the surface of A, forming stable “core-shell” hetero-clusters [55,56]. When A and B possess charges of the same sign, their mixture is often colloidally stable, forming a new, binary colloid. In this case, one can effectively realize the A-B hetero-aggregation [47,48,50,51,53,57,58,59,60,61], by introducing electrolytes to screen the electrical double layer, adding bonding molecules or high molecular weight polymers to cause depletion aggregation, or varying pH to neutralize pH-sensitive charges. However, in most of these cases, it is rather difficult to control the hetero-aggregation processes to realize uniform and random distribution of the fillers inside the polymer matrix, while avoiding aggregation among the fillers.

Toward this aim, a new technique has been developed recently, which is based on intense shear-driven aggregation of binary colloids to uniformly and randomly distribute fillers into a polymer matrix and to avoid aggregation among the fillers [48,50]. Let us consider that the B NPs are the dominant polymeric component, being the elements eventually forming the matrix, and A NPs are the fillers to be distributed. The first key feature is that the binary colloids must be very stable at rest, thus warranting initial homogeneous mixing of A and B NPs at nanoscales. The second key feature is that the nanocomposite materials are generated through (purely) intense shear-driven aggregation of the binary colloids, without using any electrolytes. The feasibility of the above procedure has been demonstrated by experiments. In this work, we review the relevant intense shear-driven aggregation and its application to binary colloids to generate the polymer matrix nanocomposites.

## 2. Intense Shear-Driven Aggregation

The intensive shear-driven aggregation has been systematically investigated in recent years for unary (single component) colloids [62,63,64,65,66,67,68,69,70,71]. For a colloidal system that is rather stable under stagnant conditions, if we drive the system to pass through a microchannel system, where a sufficiently intense shear force is generated, the particles may overcome the repulsive interaction energy barrier, $U_{T,\max}$, leading to aggregation, which was first demonstrated in diluted dispersions [72,73]. When the particle volume fraction (*φ*) reaches a certain value, the clusters formed under the intense shear would connect to form a space-spanning network, resulting in a solid-like gel.

### 2.1. Main Phenomena Observed Experimentally

**Figure 1:**
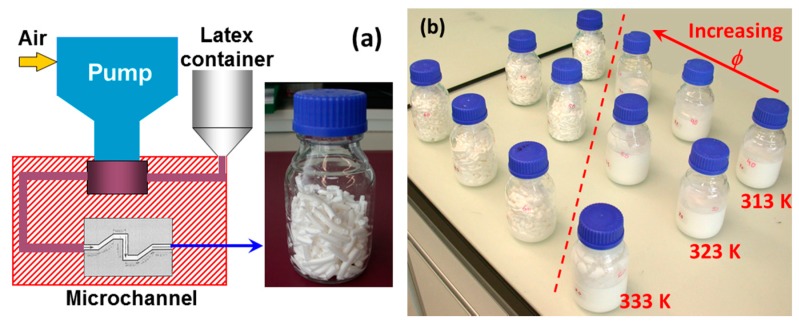
A z-shaped microchannel (z-MC) setup (**a**) for conducting intense shear-driven aggregation/gelation of colloidal dispersion, and (**b**) intense shear-driven transition from liquid-like colloids to solid-like gels at the shear rate, $\dot{\gamma }$ = 1 × 10^6^ s^–1^, at different particle volume fractions (*φ*) and different operation temperatures. Reproduced with permission from [65,74]. Copyright (2010) American Chemical Society and (2014) Elsevier.

A schematic setup for conducting intense shear-driven aggregation, using a commercial device, HC-5000 homogenizer (Microfluidics, Westwood, MA, USA), equipped with a z-shaped microchannel (z-MC), is shown in Figure 1 a. When a liquid-like colloid is forced to pass through the z-MC at an extremely high shear rate (e.g., $\dot{\gamma }$ = 1 × 10^6^ s^−1^), a solid-like (gel) transition occurs if *φ* is large enough (typically > 15%), as demonstrated in Figure 1b. Note that the cylindrical shape of the gel inside the bottles results from the tube connected to the outlet of the z-MC. There are important features for this shear-driven process. First, no additives are needed during the process, thus avoiding contamination of the final products. Second, the process can operate continuously, which is crucial for large scale industrial production.

The intense shear-driven aggregation kinetics and the time evolution of the cluster morphology have been studied using a polystyrene colloid, which has been forced to pass through the above mentioned z-MC many times, in the low range of *φ*, where, instead of gelation, only shear-driven aggregation occurs [71]. It was found that in this case a colloidal system after passing through the z-MC is composed of two distinct classes of clusters: Class 1, which is mainly composed of primary particles with small amount of small clusters made of two and three primary particles, referred to as doublets and triplets, respectively, and Class 2, which are big clusters with an average size at least two orders of magnitude larger than the primary particles, thus constituted of 10^3^–10^4^ primary particles [62,65,66]. The size and morphology of Class 2 clusters are controlled by breakage and restructuring induced by the intense shear, i.e., the size decreases as the shear rate increases. The size distribution is typically rather uniform.

**Figure 2:**
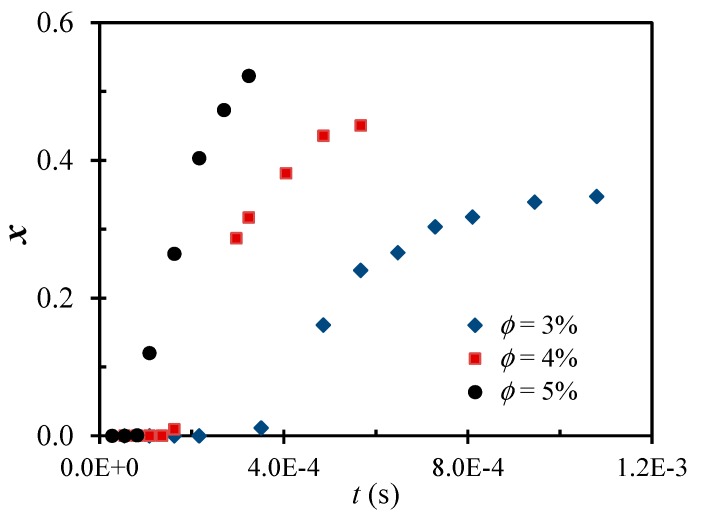
Time evolution of the conversion of polystyrene NPs to big clusters through the intense shear-driven aggregation at a fixed shear rate, $\dot{\gamma }$ = 1.5 × 10^6^ s^−1^, and various particle volume fractions. Reproduced with permission from [71]. Copyright (2014) American Chemical Society.

Figure 2 shows the shear-driven aggregation of the polystyrene colloid in the z-MC, particularly, the evolution of the primary particle conversion (*x*) to big (Class 2) clusters as a function of time. Note that the time here is a cumulative (residence) time from forcing the same colloid to repeatedly pass through the z-MC many times. It is seen that the *x* evolution is typically composed of three stages: induction, sharp increase and slow increase to reach a plateau. In the induction stage, the *x* value is negligible. In the stage where *x* increases sharply with the pass number (i.e., the shearing time), the average size of Class 2 clusters increases also sharply, often characterized by an overshooting. In the last stage where the *x* value increases slowly and eventually reaches a plateau, the average size of Class 2 clusters decreases to also reach a plateau. The fractal dimension of Class 2 clusters increases with the shearing time from the initial value of 2.40±0.05 to reach 2.80±0.05. Thus, the final clusters generated by the intense shear are typically rather compact.

It should be mentioned that the plateau value of *x* reached at large shearing times at each particle volume fraction cannot be explained at the present stage, which happened also for the intense shear-driven aggregation of the other colloidal systems [50]. In principle, as a second-order kinetics, the intense shear-driven aggregation of the primary particles to big clusters should continue until reaching 100% conversion. To understand this behavior, new experiments have been designed and the investigation is continuing in our lab.

### 2.2. Theoretical Background of the Intense Shear-Driven Aggregation

A theory has been developed to better understand the shear-driven event [63,67,69]. We start with the simplest case, the doublet formation. For interactive particles embedded in a linear velocity field, the stationary particle concentration field *c*(*x*) can be written following the Smoluchowski equation

(1) $$\nabla \left\{\frac{D}{k_{B}T}\left[-\nabla U_{T}(x)+3\pi \eta av(x)\right]-D\nabla \right\}c(x)=0$$

where *D* is the mutual diffusion coefficient of the particles (*D* = 2*D*_0_*G*(*x*), where *D*_0_ is the diffusion coefficient of an isolated particle, and *G*(*x*) is the hydrodynamic correction for viscous retardation), *k*_B_ is Boltzmann’s constant, *T* is the absolute temperature, *U*_T_(*x*) is the colloidal interaction energy, η is the viscosity of the solvent, *a* is the particle radius, and *v*(*x*) is the flow velocity. To solve Equation (1), the boundary conditions for the irreversible shear-driven aggregation are given by

(2) $$\begin{array}{l} c\left(x\right)=0\;\mathrm{at}\;x=0,\\ c\left(x\right)=c_{0}\;\mathrm{at}\;x=\delta /a \end{array}$$

where the δ value can be determined from the boundary layer approximation [63]

(3) $$\delta /a=\sqrt{\left(1/\kappa a\right)/Pe}$$

where *κ* is the Debye length, and Peclet number, *Pe*, is defined as,

(4) $$Pe\;=\;\frac{3\pi \eta \dot{\gamma }a^{3}}{k_{B}T}$$

with $\dot{\gamma }$ the shear rate. From Equation (1), the derived kinetic constant or kernel, *k*_1,1_, for the aggregation between two particles forming a doublet can be expressed as,

(5) $$k_{1,1}=\frac{8\pi Dac_{0}}{\int_{0}^{\delta /a}\frac{dx}{G(x){\left(x+2\right)}^{2}}\exp\int_{\delta /a}^{x}dx\left(\frac{1}{k_{B}T}\frac{dU_{T}(x)}{dx}+Pe{\tilde{v}}_{r}\right)}$$

where ${\tilde{v}}_{r}$ is the effective velocity for aggregation. The *k*_1,1_ values predicated from Equation (5) have been verified by comparing them with those obtained from numerical simulations of the full convective diffusion equation, Equation (1) [63].

After proper simplification and approximation in the frame of the DLVO (Derjaguin-Landau-Verwey-Overbeek) interactions, Equation (5) can reduce to the following Arrhenius form

(6) $$k_{1,1}\approx 8\pi Dac_{0}{\left(Pe-\frac{U_{T,\max}^{\prime \prime }}{k_{B}T}\right)}^{1/2}\exp\left(-\frac{U_{T,\max}}{k_{B}T}+2\alpha Pe\right)$$

where $U_{T,\max}^{\prime \prime }<0$, and α is a geometrical parameter. From Equation (6), it can be clearly seen that at small *Pe* values, the exponent can be negative so that the aggregation rate is rather small. As the *Pe* value increases, the collision rate increases, and once *Pe* increases to a certain critical value, *Pe_cr_*, which can be defined by setting the exponent in Equation (6) equal to zero as

(7) $$Pe_{\mathrm{cr}}=\frac{U_{T,\max}}{2\alpha k_{B}T}$$

the shear force (*Pe*) plays a prominent role. In particular, when *Pe* << *Pe_cr_*, the colloidal interaction barrier (*U*_T__,max_) plays the dominant role, and the aggregation rate (*k*_1,1_) increases as *U*_T__,max_ decreases, corresponding to the Brownian motion controlled aggregation. When *Pe* >> *Pe_cr_*, the shear force takes over the dominant role, and the effect of *U*_T__,max_ becomes negligible. The *k*_1,1_ value increases exponentially with *Pe*, corresponding to the shear controlled regime.

It should be particularly noted that from Equation (4), *Pe* is proportional to the radius of the particles, *a*, to a power of 3, indicating that the particle or cluster size has substantial influence on the aggregation rate. During the shear-driven event, the formed doublets progressively grow to larger clusters. Then, even though initially one has *Pe* << *Pe*_cr_, as the cluster size increases progressively with time to reach a critical radius, *a*_cr_, the situation, *Pe* >> *Pe*_cr_, occurs, leading to an exponential increase in *k*_1,1_, thus, self-acceleration. The critical cluster radius, *a*_cr_ is given by

(8) $$-\frac{U_{T,\max}}{k_{B}T}+2\alpha \frac{3\pi \eta \dot{\gamma }a^{3}}{k_{B}T}=0\;\Rightarrow \;a_{\mathrm{cr}}={\left(\frac{U_{T,\max}}{6\pi \eta \alpha \dot{\gamma }}\right)}^{1/3}$$

The above self-accelerating aggregation predicted by the theory is in excellent agreement with the experimental results in Figure 2. At each particle volume fraction, the induction stage corresponds to the time needed to reach the critical value, *a*_cr_ and then after the radius of the clusters has reached *a*_cr_, the conversion to big clusters increases sharply, i.e., the aggregation accelerates. The *a*_cr_ value has been identified experimentally and confirmed to increase as the shear rate decreases [71].

## 3. Applications of the Shear-Driven Aggregation to Binary Colloids

**Figure 3:**
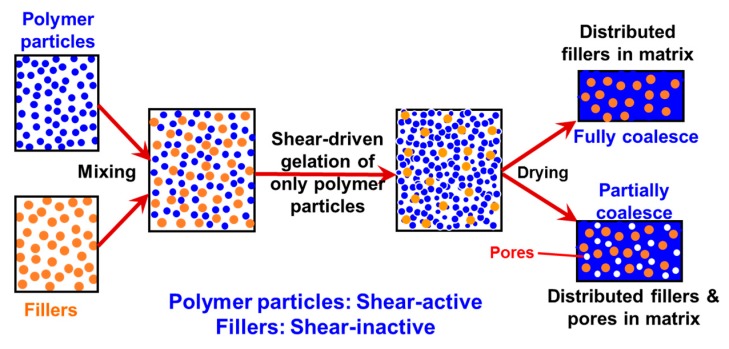
Design concept for fabrication of nanocomposites via purely shear-driven aggregation/gelation of binary colloids.

Recently, in the frame of preparation of nanocomposites where the fillers have to be uniformly and randomly distributed inside a polymer matrix, a general methodology has been developed [48,50], which is purely based on the intense shear-driven aggregation discussed above but starting with binary colloids, without using any additives. The setup used for the shear-driven aggregation of the binary colloids is the same as that sketched in Figure 1a. The design concept is shown in Figure 3 and described as follows:

(1) It starts with two colloids: fillers (A) NP dispersion and polymer (B) NP dispersion, both of which have the same sign of charges;
(2) When the two colloids mix, a stable binary colloid is formed, where A and B NPs are homogeneously distributed at nanoscales;
(3) The polymer (B) is the dominant colloid, and the volume fraction of the fillers (A) is smaller;
(4) The colloidal interactions are controlled such that the B (polymer) NPs are unstable under the used shear rate (referred to as shear active) and can aggregate to form clusters or gels. Instead, the A (fillers) NPs are very stable under the same shear rate and the shear-driven aggregation does not occur, referred to as shear-inactive.

Thus, when the binary colloid passes through the z-MC as shown in Figure 1a at an extremely high shear rate and at a significantly high particle volume fraction (typically *φ*_B_ > 15%), the binary colloid will be converted to a solid-like gel after passing through the z-MC. It should be particularly noticed that since the residence time of the colloid in the z-MC is very short, in μs scales, the extremely fast gelation of the B NPs can basically freeze the initially distributed A NPs (fillers) inside the formed gel. It is this frozen mechanism that avoids the possibility of aggregation among the A NPs, being the key novelty of the proposed methodology.

After the solid-like gel is formed and dried at a temperature larger than the melting point of the polymer (B), the B NPs can fully melt to form a matrix where the fillers (A) NPs are uniformly and randomly distributed with negligible aggregation among the A NPs. If one properly controls the drying temperature such that the polymer particles are only partially coalesced, there is the possibility of having desired pores inside the nanocomposites. In the following subsections, we show two examples, where we demonstrate that, based on the design concept in Figure 3, the intense shear-driven aggregation of binary colloids can indeed successfully distribute fillers homogeneously inside the polymeric NP gels.

### 3.1. Distribution of PMMA Particles into PS NP Matrix

The first example is a model system where poly-methyl methacrylate (PMMA) NPs are distributed in the matrix of polystyrene (PS) NPs [48]. Two types of colloids, the PMMA and PS NP aqueous dispersions, have been first synthesized separately. The PS NPs are charged with the fixed negative charges, –OSO_3_– and can undergo the shear-driven aggregation (shear-active), at a shear rate of $\dot{\gamma }$ = 1.5 × 10^6^ s^−1^. The PMMA NPs are also charged with –OSO_3_– In addition, the PMMA particles possess certain hydrophilicity, because of the surface ester groups from the MMA monomers, which favor the tendency of forming ordered water layers [75]. Thus, the PMMA particle surface possesses substantial short-range, repulsive hydration forces. These non-DLVO forces together with the DLVO interactions from the charges provide an extremely high energy barrier and very shallow primary minimum [74], such that aggregation among the PMMA particles under the same shear rate does not occur. For example, the prepared PMMA colloid with the radius of the primary particles, *a*_PMMA_ = 75 nm, does not undergo any aggregation after passing through the z-MC at $\dot{\gamma }$ = 1.5 × 10^6^ s^−1^, even for more than 20 times at a particle volume fraction larger than 20%.

**Figure 4:**
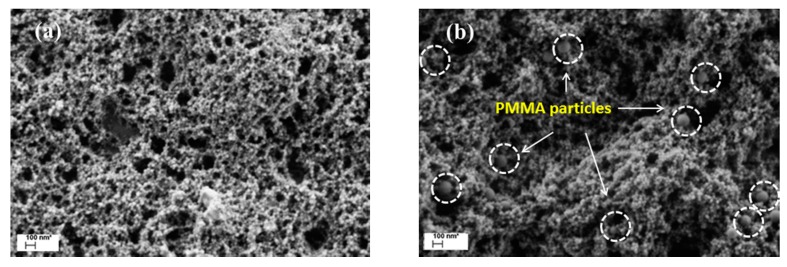
SEM pictures of the clusters formed after shear-driven aggregation of (**a**) pure PS dispersion and (**b**) the PMMA + PS binary dispersion. Reproduced with permission from [48]. Copyright (2016) The Royal Society of Chemistry.

Then, the PS and PMMA NP colloids are mixed to form a new binary colloid, which is stable at rest. When this binary colloid is forced to pass through the z-MC, the shear-driven aggregation/gelation can also occur, and the formed gels are composed of both the ‘shear-active’ PS and ‘shear-inactive’ PMMA particles. Moreover, in the case of low particle volume fractions where no gelation occurs, the aggregated binary system, similar to the unary system, is also composed of two distinct classes of clusters: Class 1, which is mainly composed of the primary PS and PMMA NPs; and Class 2, which are big clusters (mainly made of the PS NPs) with an average size at least two orders of magnitude larger than the primary NPs. Figure 4 compares typical morphology of the clusters formed after shear-driven aggregation of the pure PS colloid and of the PMMA/PS (*a*_PMMA_ = 75 nm, *a*_PS_ = 21 nm) binary colloid. It is seen that the general morphology is rather similar in Figure 4a,b, because in both cases it results from aggregation of PS NPs. The only difference is that in Figure 4b the PMMA particles are uniformly and randomly distributed (captured) within the clusters of the PS particles.

**Figure 5:**
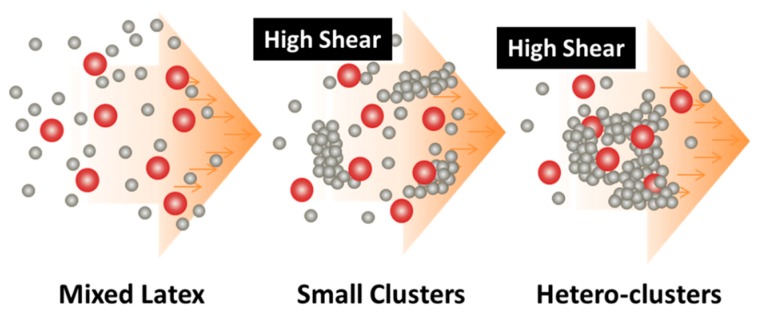
A schematic representation of the shear-driven aggregation mechanism of the PS/PMMA binary colloid, where the small PS NPs are shear active, while the PMMA NPs are shear inactive. Reproduced with permission from [48]. Copyright (2016) The Royal Society of Chemistry.

In addition, it was found that the presence of the PMMA NPs does not affect significantly the shear-driven aggregation kinetics of the PS NPs nor the average cluster size at low volume fractions, and the PMMA NPs behave as inert fillers. Therefore, the above results confirm the proposed shear-driven aggregation mechanism of the binary colloids. As schematically shown in Figure 5, since the shear-driven aggregation of the shear-active PS NPs takes place in micron seconds in the z-MC, the initially distributed PMMA NPs in the binary colloid, though shear-inactive, do not have time to escape from the PS aggregation process and are captured inside the clusters. The PMMA NP capture efficiency may reach 100% at low PMMA volume fractions, but it can be significantly lower at high PMMA volume fractions, particularly when the PMMA/PS size ratio is very large. On the other hand, if the volume fraction of the shear-active PS NPs in the binary system is high enough, such that the shear-driven gelation occurs after the binary colloid passing through the MC just one time, the PMMA NP capture efficiency can naturally reach 100%.

### 3.2. Distribution of GO Sheets into PVDF NP Matrix

**Figure 6:**
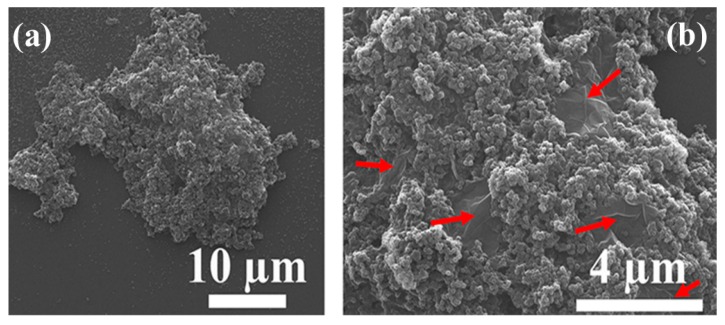
SEM images of the clusters formed by the GO/PVDF binary colloid after passing through the z-MC one time: (**a**) typical shape of the clusters and (**b**) the dispersed GO sheets inside the clusters, as indicated by the red arrows. Reproduced with permission from [50]. Copyright (2016) The Royal Society of Chemistry.

The same methodology has been further verified by Sheng et al. [50], for a binary colloidal dispersion composed of graphene oxide (GO) sheets and poly-vinylidene fluoride (PVDF) NPs. In this case, the GO sheets are fillers. They are shear-inactive, because there are many oxygen-containing groups (e.g., –OH and –COOH) on their edges and epoxide groups on their basal planes. These groups are rather hydrophilic, leading to the GO sheets being extremely stable under the intense shear rate. Instead, the (dominant component) PVDF NPs are shear-active, because of extreme hydrophobicity of the polymer. After passing the binary dispersion through the z-MC device at an extremely high shear rate (~10^6^ s^−1^), aggregation/gelation of the shear-active PVDF NPs occurs, leading to uniform capture and distribution of the shear-inactive GO sheets inside the PVDF NP matrix, as demonstrated by the SEM pictures in Figure 6. Therefore, this example demonstrates again that through the intense shear-driven aggregation of binary colloids, one can effectively distribute uniformly and randomly the shear inactive fillers inside a polymer matrix, leading to well-defined nanocomposites.

**Figure 7:**
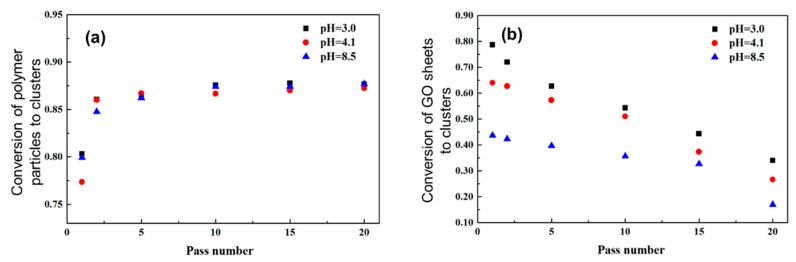
Conversion of the polymer (PVDF) particles (**a**) and the GO sheets (**b**) to big clusters, as a function of the number of passes. The volume fraction of PVDF NPs = 2.9%, and the volume fraction of the GO sheets with respect to the polymer = 3.0%. Reproduced with permission from [50]. Copyright (2016) The Royal Society of Chemistry.

In the case of low initial particle volume fractions where only partial aggregation-instead of complete gelation-occurs after the binary colloid passes through the z-MC one time, it is observed that the GO/PVDF NP ratio inside the formed Class 2 clusters is lower than that in the initial binary colloid. Moreover, if one allows the aggregated system to repeatedly pass through the z-MC, although the conversion of the PVDF NPs to Class 2 clusters increases with the pass number, as shown in Figure 7a, the amount of the captured GO sheets inside the clusters decreases monotonically with the pass number, as reported in Figure 7b. This arises because of two main reasons. First, the GO sheets are shear inactive and only captured (instead of aggregated) inside the PVDF NP clusters during the extremely fast shear-driven aggregation. Second, along the shear-driven aggregation in the z-MC, there are also cluster breakage and restructuring, and these processes provide the opportunity to the captured GO sheets to escape from the Class 2 clusters. In addition, it can be observed from Figure 7b that the fraction of the GO sheets inside the clusters decreases as pH increases. This is considered to be related to some specific interactions induced by the H⋅⋅⋅F halogen bonding between the –CF_2_– groups of the PVDF NPs and the –COOH groups on the GO sheet [76,77]. In particular, the –COOH groups on the GO sheets would be progressively deprotonated as pH increases, and this would reduce the H⋅⋅⋅F halogen bonding interactions, thus promoting the escape of the GO sheets from Class 2 clusters during the shear-driven aggregation/breakage. This phenomenon indicates that during the preparation of the nanocomposites through the shear-driven aggregation of binary colloids, one should also consider the roles played by the various interactions among the functional groups present on the surface of the two colloidal identities.

On the other hand, in Figure 7a, the conversion of the PVDF NPs to Class 2 clusters, *x*_p_, does not change significantly with pH. This arises because the PVDF NPs are shear-active, and their aggregation is purely shear-controlled [63,65]. In the shear-controlled regime, the evolution of the aggregation is independent of the surface charge or potential and governed by the value of the Peclet number, *Pe*, defined by Equation (4).

The above results indicate that to effectively capture the GO sheets inside the PVDF NP clusters, one should let the binary colloid pass through the z-MC only one time, instead of many times. As mentioned above, if a solid-like gel of the binary colloid can be formed after passing through the z-MC just one time, all the GO sheets can be captured inside the gel, leading to 100% capture efficiency. Such an observation is particularly important, as an essential guideline, in practical applications of the developed methodology to form nanocomposites.

In addition, it should be noted again that for this binary colloid, the conversion of the PVDF NPs to Class 2 clusters in Figure 7a also reach a plateau value, which is far below 100% conversion, as noticed in the unary colloid in Figure 2.

## 4. Concluding Remarks and Perspectives

In this short review, we have presented recent advances in our knowledge about the aggregation of unary and binary colloids. We have introduced the aggregation and gelation of colloids purely based on intense shear force and the relevant theory and experimental validation. Then, we have described how to apply the intense shear-driven aggregation technique to binary colloids to generate nanocomposite materials, where A NPs as fillers are uniformly and randomly distributed inside a matrix of B polymer NPs. We have also illustrated the advantages of the developed methodology compared with the other common methods.

The feasibility of the proposed methodology has been demonstrated using two stable binary colloids: one is composed of PMMA NPs as the fillers and PS NPs as the matrix, and another of GO sheets (fillers) and PVDF NPs. In the first case, the PS NPs alone undergo the shear-driven aggregation (shear-active), while the PMMA fillers alone do not (shear-inactive). It was observed that the shear-driven aggregation of the PMMA/PS binary colloids can occur, and the formed clusters or gels are composed of both components. More importantly, the PMMA fillers are uniformly and randomly distributed inside PS NP matrix. Similar results were also obtained in the case of GO/PVDF binary systems, and the gelation of the shear-active PVDF NPs is able to uniformly capture and distribute the shear-inactive GO sheets inside the PVDF NP gel matrix. All the results confirm the feasibility of the proposed methodology.

The shear-driven aggregation mechanism of the binary colloids for the filler capturing and distribution has been illustrated, which would help broaden the means of fabricating nanocomposites through the binary colloidal route. Typical applications include incorporation of fillers (e.g., graphene nanosheets, clays, or silica or oxide NPs) into the polymer NPs matrix to generate highly electronically conductive materials, high-performance gas barrier composite membranes, composite separators for lithium-ion batteries, etc. Studies regarding these applications have been started in our groups, and the preliminary results for the properties of the generated nanocomposites are very encouraging.
